# Three Cases of Molluscum Contagiosum

**Published:** 1878-12

**Authors:** Roswell Park

**Affiliations:** Chicago, Ill.


*Three Cases of Molluscum Contagiosum.*

In the May number of this journal is a paper by Dr. G. H. Fox, of New York, entitled, "A Clinical Study of Molluscum Contagiosum," with a report of twenty-five cases. As it has fallen to my lot to have seen, within a year, three cases of this rather rare complaint, I have thought it worth while to put them on record, especially as two of them exhibited peculiar features.

Case I. A little girl was brought to the Eye and Ear Infirmary last winter with a single molluscum near one external canthus. No history of contagion was obtained. It was removed by Prof. Holmes, and the case disappeared from observation.

CASE II. J. M., æt. 41, came to my dispensary clinic last January for advice and treatment. He said that several months before the present visit he noticed several spots about his neck which were tender to the touch, and whose presence annoyed him. Soon there was elevation of the skin, and after a while he noticed at times a discharge from little openings in the elevated patches. Without causing positive pain, there was a constant feeling of discomfort, and the contact of his clothes with the parts seemed to irritate. Upon examination, several flat, sessile tumors were seen scattered about the neck, especially on the sides, varying from 3 m. m. to 3 c. m. in size. About the center of each of the smaller ones was apparently an opening, while the larger presented several openings, from which either protruded or could be easily expressed the characteristic whitish, curdy contents. No specific history could be obtained, nor anything which could throw any light upon the trouble; he was apparently, otherwise, in excellent health.

An ointment of iodine was prescribed, and no prognosis ventured upon. At a subsequent visit the application of iodine seemed to have been of some benefit, and I desired to give it a fair trial before resorting to the knife or cautery. I sought the counsel of Profs. Davis and Andrews, however, and they both recommended final resort to operative measures. I explained this to the patient, but he never reappeared for further treatment.

CASE III. W. J. C., æt. 18, American, applied also, during August, at the dispensary for relief. He presented a very cachectic appearance. He had been under the care of different physicians of the city. He stated that he had had diabetes for about seven months. Examination of the urine confirmed this statement. Said he had lost some 1.5 kilog. within a year. The average amount of urine passed during the 24 hours was about twelve litres. Every winter he has had an itching of the skin, without eruption, unless he scratched himself (urticaria ?), but last winter was annoyed more than usual by this trouble. During last winter, also, wart-like growths appeared scattered over his legs, and since then have increased in number and size. He had, at time of examination, from fifty to seventy-five of these growths on each leg below the knee, a few on the thighs, and one or two on the neck and chest. They presented the typical appearance, but were rather sessile, of size varying from that of a pea to that of a dime; one or two of them breaking down and forming ulcers. He stated positively that some of them had from time to time disappeared, while other and fresh ones made their appearance. The mollusca were all quite tender, and occasionally painful. In addition to the above, upon examination of the genitals, a marked condition of phimosis was found. Opportunity was not afforded to trace any possible connection between this latter difficulty and the diabetic cachexia. There was no history or indication of syphilis, either hereditary or acquired. He was put on tonic treatment, and circumcision strongly recommended; but the patient declined the latter measure, and disappeared from observation.

It is very unfortunate that these cases could not have been watched and their progress noted, but the mere suggestion of operative relief seemed to frighten them away. Case III. was seen with me by Dr. Hyde, who approved the treatment and recommendations.

By this simple report I have endeavored only to put these cases on record, and have conscientiously avoided saying anything about their pathology. In none of them could it be learned that others of the family or neighbors were similarly affected.

ROSWELL PARK, M. D.

CHICAGO, ILL.

---

THE HOSPITALS OF PRUSSIA IN 1877. According to the statistical report for 1877, Prussia had 643 public general hospitals, with 27,633 beds; 244 private general hospitals, with 7,906 beds; 53 public insane asylums, with 12,791 beds; 73 private insane asylums; 7 public eye infirmaries, with 167 beds; 25 private eye institutes, with 484 beds; and 31 public and 57 private lying-in hospitals.

